# Family and Psychosocial Functioning in Bipolar Disorder: The Mediating Effects of Social Support, Resilience and Suicidal Ideation

**DOI:** 10.3389/fpsyg.2021.807546

**Published:** 2022-01-28

**Authors:** Wenbo Dou, Xueying Yu, Hengying Fang, Dali Lu, Lirong Cai, Caihong Zhu, Kunlun Zong, Yingjun Zheng, Xiaoling Lin

**Affiliations:** ^1^School of Nursing, Sun Yat-sen University, Guangzhou, China; ^2^Third Affiliated Hospital of Sun Yat-sen University, Guangzhou, China; ^3^Xiamen Xianyue Hospital, Xiamen, China; ^4^Guangdong Provincial People’s Hospital, Guangzhou, China; ^5^Guangzhou Brain Hospital, Guangzhou Medical University, Guangzhou, China

**Keywords:** bipolar disorder, family functioning, psychosocial functioning, social support, resilience, suicidal ideation

## Abstract

Patients with bipolar disorder (*BD*) may experience family dysfunction, which might result in worse psychosocial functioning through environmental and psychological factors. Research investigating the mediating role of social support, resilience and suicidal ideation on family and psychosocial functioning in BD is rare. The study aims to explore the predicting and mediating effects of social support, resilience and suicidal ideation on family and psychosocial functioning in BD patients. Two hundred forty-six patients with BD and sixty-nine healthy controls were recruited. The Family Assessment Device (*FAD*), Functioning Assessment Short Test (*FAST*), Social Support Rating Scale (*SSRS*), Connor-Davidson Resilience Scale (*CD-RISC*) and Beck Scale for Suicide Ideation (*BSI*) were used to assess family functioning, psychosocial functioning, social support, resilience and suicidal ideation, respectively. Bipolar patients exhibited worse family and psychosocial functioning than healthy controls. Family functioning, social support, resilience and suicidal ideation significantly predict psychosocial functioning in the bipolar group. Social support, resilience and suicidal ideation indirectly mediate the effect of family functioning on psychosocial functioning in bipolar patients. Cross-sectional design and mixed sample including acute and remitted stages. Treatments for patients with bipolar disorder should be combined with family strategies that are formulated to improve psychosocial functioning. An emphasis should be placed on enhancing social support and resilience while paying attention to suicidal ideation.

## Introduction

Bipolar disorder (BD) is a lifelong refractory psychiatric mental illness that has a high episode frequency, comorbidity and poor functional outcomes (Du Rocher et al., 2008; Sole et al., 2018; Carvalho et al., 2020). Almost all patients with BD are challenged to maintain family relationships while managing severe psychosocial functioning. Impairments in family functioning are one of the most functional impairments in bipolar disorder, and poorer family functioning is associated with impaired psychosocial functioning (Du Rocher et al., 2008; MacPherson et al., 2018). Evidence has shown that family-based psychosocial interventions can reduce the number of hospital admissions and decrease the risk of relapse (West and Cosgrove, 2019). Research on family and psychosocial functioning in serious mental illness has gained increasing interest in recent years.

Bipolar disorder patients and their family members have been characterized by high levels of expressed emotion, the absence of family cohesion and family adaptability and significant inadequate family interpersonal relationships (West and Cosgrove, 2019; Zhang et al., 2019). All these features of bipolar families result in lower perceived family and social support from surroundings and are associated with difficulties establishing intimate relationships. Family functioning as a risk or a protective factor plays a critical role throughout the lives of BD patients (Chang et al., 2001). For instance, lower family cohesion and adaptability and higher conflict could predict poor mood symptoms in patients with BD (Sullivan et al., 2012). However, family-focused therapy to promote emotional stability improves family functioning by enhancing the problem solving and communication abilities of BD patients (Miklowitz and Chung, 2016). Patients with bipolar disorder always experience impairment of psychosocial functioning during observation of family relationships and life satisfaction (Perlis et al., 2009).

Notably, family variables have been demonstrated to serve as moderators and mediators of psychosocial outcomes in BD patients (Sullivan et al., 2012; MacPherson et al., 2018). Nevertheless, Stapp et al. (2019) demonstrated that parental BD predicts high family conflict and poor family environment, and mothers with BD require psychosocial support to address family functioning. Parental psychosocial functioning mediates the correlation between clinical symptoms and family dysfunction (Shalev et al., 2019). Improvement in symptomatic remission does not mean the recovery of psychosocial functioning (Karow et al., 2012); environmental factors, such as family functioning and social reintegration, cannot be ignored (Sullivan et al., 2012; Guerrero-Jimenez et al., 2021). The relations between family and psychosocial functioning are likely bidirectional. Psychosocial functioning interventions are beneficial in improving family conflict, enhancing cohesion and moderating family adaptability in bipolar disorder patients (O’Donnell et al., 2020). However, research analyzing the correlations between family and psychosocial functioning in patients with bipolar disorder is rare. Previous research has always paid exclusion attention to pharmacotherapy in patient illness episodes without consideration of their familial circumstances and the adaptive capacity of considerable adversity (Miklowitz and Chung, 2016). Thus, the correlation between family and psychosocial functioning warrants further investigation.

The current findings by Dunne et al. (2019) suggest that the support of family, friends and partners may contribute to personal recovery and psychosocial functioning in BD patients and help individuals build resilience and cope with adverse environments effectively (Zhou et al., 2019). Structural equation modeling illustrated that social support has an irreplaceable effect between clinical symptoms and household poverty, and better social support indicate higher household income and lower caregiving burden (Yu et al., 2020). Dou et al. (under review) reported that higher social support predicted better family and psychosocial functioning, and social support also played a moderating role in the relationship between family functioning and psychosocial functioning in BD patients. A strong relationship was noted between impaired family functioning and inadequate social support in depression patients (Wang and Zhao, 2012). Additionally, social support had a mediating and moderating effect between childhood abuse and subsequent outcomes (Sperry and Widom, 2013). However, the relationship among family functioning, social support and psychosocial functioning in patients with BD also requires further exploration.

Resilience refers to the approach to positive adaptation when confronting stress, trauma, family tragedy or significant adversity that would be expected to cause acute sequelae (Cicchetti, 2010). Individuals and families are able to respond successfully to disadvantages and persistent challenges and to recover and expand through resilience, which was proposed in the family resilience framework (Walsh, 2003). The present study exemplified that better performance in family functioning indicated greater resilience and predicted better mental health in hemodialysis patients (Kukihara et al., 2020). Resilience and life satisfaction are partially mediated by perceived social support in substance use disorder (Yang et al., 2020). Higher resilience is associated with better psychosocial functioning, which has been investigated in clinically stable BD outpatients (Mizuno et al., 2016). Hence, the finding explored by Kim et al. (2013) suggest that a significant relationship exists between impaired psychosocial functioning and a lower level of resilience in individuals at ultrahigh risk for psychosis. The level of resilience in BD patients is lower than that in healthy controls, even in the euthymic period (Lee et al., 2017). However, few studies have focused on examining the relationship among resilience, family functioning and psychosocial functioning in patients with BD.

Furthermore, the role of suicidal ideation between family and psychosocial functioning in BD has been poorly investigated. Approximately 59% of patients with BD have suicidal ideation, and this proportion is 20–30 times that of the general population (Abreu et al., 2009; Pompili et al., 2013). Excessive expressed emotion, reduced family cohesion and increased family conflict are associated with increased suicidal ideation in patients with BD (Weinstein et al., 2015; Berutti et al., 2016). Young adults with enduring suicidal ideation often have a heightened risk of psychosocial dysfunction (Steinhausen and Metzke, 2004). Our previous research failed to explore the correlation between suicidal ideation and psychosocial functioning (Luo et al., 2020). It is necessary to further clarify how suicidal ideation impacts family functioning and psychosocial functioning, which will contribute to accurate intervention measures in family and psychosocial functioning and reduce suicidal attempts in BD patients.

To the best of our knowledge, this is the first study to explore the mediating effects of social support, resilience and suicidal ideation on the relationship between family and psychosocial functioning in BD patients. The study aims (i) to compare family functioning, psychosocial functioning, social support, resilience and suicidal ideation between bipolar patients and healthy controls; (ii) to evaluate the associations among family functioning, social support, resilience, suicidal ideation and psychosocial functioning in BD patients; (iii) to identify potential predictors of psychosocial functioning in bipolar patients; and (iv) to investigate the mediating effects of social support, resilience and suicidal ideation on family and psychosocial functioning in bipolar patients.

## Materials and Methods

### Participants

A cross-sectional design was used in the present study. Patients enrolled in this study were approved by the psychiatric inpatient department and outpatient department of Xiamen Xianyue Hospital, the Affiliated Brain Hospital of Guangzhou Medical University and the Third Affiliated Hospital of Sun Yat-sen University, Xiamen and Guangzhou Cities, China between April 2019 to December 2019 and September 2020 to April 2021. Two psychiatrists are responsible for the diagnosis and clinical states, using the Structured Clinical Interview for *DSM-V* Axis I Disorders, Clinical version (*SCID-CV*) in conjunction with the Young Mania Rating Scale (*YMRS*) (Young et al., 1978) and the 17-item Hamilton Depression Rating Scale (*HDRS-17*) (Hamilton, 1960). Patients’ exclusion criteria included current or lifetime diagnosis of active psychotic symptoms or intellectual disability (Wechsler Adult Intelligence Scale score <70); dementia, substance or alcohol abuse within one year; head injury; electroconvulsive therapy (*ECT*) in the last year; engagement in any structured psychological intervention that might affect cognitive functioning within the last 2 years; and other physical or neurological illness or an unstable medical disease condition. Finally, a total of 246 patients with a mean age of 28.37 (*SD* = 11.92) years old, including manic and hypomanic as well as depressed and euthymic states, were included in this research. No patient was drug-free, and valproate, lithium and antipsychotics (including quetiapine, olanzapine and risperidone, etc.) were the three most frequently used drugs (see Table 1).

**TABLE 1:** Demographic, clinical and pharmacological characteristics in patients with bipolar disorder and healthy controls.

Variables	Patients with bipolar disorder (*n* = 246)		Healthy controls (*n* = 69)		ANOVA	
			
	*Mean*	*SD*	*Mean*	*SD*	*F*	*p*
Age (years)	28.37	11.92	31.3	9.29	11.048	0.059
Body mass index, (BMI, kg/m^2^)	22.43	4.11	22.69	4.54	0.036	0.637
Waist hip ratio (WHR)	0.86	0.09	0.83	0.07	13.340	0.035*
HDRS-17 score	9.57	7.68	2.00	2.31	58.191	<0.001**
YMRS score	6.68	5.73	1.04	2.50	47.491	<0.001**
Age at onset (years)	21.22	8.42	–	–	–	–
Age at treatment (years)	22.89	8.40	–	–	–	–
Duration of illness (years)	7.56	8.01	–	–	–	–

	***N***	***%***	***N***	***%***	***χ^2^*/*t***	***p***

Gender (Male/Female)	90/156	36.6/63.4	27/42	39.1/60.9	0.150	0.699
Race (Han/Others nations)	242/4	98.4/1.6	67/2	97.1/2.9	0.467	0.494
Marital status (single/non-single)	163/83	66.3/33.7	42/27	60.9/39.1	0.689	0.407
Occupational status (yes/no)	50/196	20.3/79.7	56/13	81.2/18.8	89.318	<0.001**
Educational level					34.766	<0.001**
≤ Primary school	72	29.3	14	20.3		
Middle school	106	43.1	10	14.5		
≥ College	68	27.6	45	65.2		
Family psychotic history (yes/no)	53/193	21.5/78.5	–	–	–	–
Past psychotic history (yes/no)	32/214	13.0/87.0	–	–	–	–
**Medications**						
Lithium	88	35.8	–	–	–	–
Valproate	106	43.1	–	–	–	–
Lamotrigine	15	6.1	–	–	–	–
Oxcarbazapine	33	13.4	–	–	–	–
Antipsychotics	203	82.5	–	–	–	–
Antidepressants	15	6.1	–	–	–	–
Benzodiazapines	88	35.8	–	–	–	–
Benzhexol	24	9.8	–	–	–	–
Propranolol	17	6.9	–	–	–	–
Others	50	20.3	–	–	–	–

*ANOVA, Analysis of Variance; SD, Standard deviation; HDRS-17, The 17-item Hamilton Depression Rating Scale; YMRS, The Young Mania Rating Scale; Others, Coenzyme Q10 Capsules; Bisoprolol Fumarate; Hydrotalcite; Omeprazole; Metoprolol; Bisacodyl; Polyene Phosphatidylcholine Capsules; Metformin.*

*p < 0.05, **p < 0.01.

Healthy individuals were recruited form two communities in Guangzhou, China, using a convenience sampling method. The healthy group was matched to the age and gender of patients of bipolar disorder. Finally, sixty-nine healthy controls (*HCs*) with a mean age of 31.3 (*SD* = 9.29) years old, had a negative history of psychiatric disease both personally and in their first-degree relatives, and the participants failed to reach the criteria of any axis mental disorder evaluated by the DSM-V Structured Clinical Interview. Additionally, participants who were pregnant or lactating were excluded. All participants communicated using Chinese and completed all assessments independently. Written informed consent was provided by each participant following a detailed explanation of the procedures. Ethical authorisation for the article, which has been obtained ethics committee approval by Sun Yat-sen University.

### Assessments

#### Demographic and Clinical Assessments

Demographic, clinical and pharmacological data were collected *via* structured interviews with the patients and/or their guardians and clinical records (see Table 1). The measurements of depression and manic symptoms were assessed using the HDRS-17 and YMRS, respectively. A psychiatrist was responsible for measuring the psychotic symptoms of patients who were blinded to the clinical and psychosocial evaluation results.

#### Family Functioning

Family functioning was assessed using the Family Assessment Device (*FAD*) (Epstein et al., 1983). The FAD is a 60-item screening self-rating questionnaire used to measure possible problems in the familial system. Each item uses a 4-level rating: range from 1 to 4 point. Higher scores on the total scale or its subscales indicated worse family functioning.

#### Psychosocial Functioning

The Functioning Assessment Short Test (*FAST*) was used to measure psychosocial functioning based on 24 items (Bonnin et al., 2016). Each item of the FAST is answered on a four-point Likert-type score ranging from 0 to 3, and the total score ranges from 0 to 72 with higher scores indicating worse psychosocial functioning (Zhang et al., 2018).

#### Social Support

The Social Support Rating Scale (*SSRS*) was used to measure the social support of patients with bipolar disorder. The SSRS is consisting of 10 items measuring the perception of subjects from subjective and objective support, and support utilization. The total score ranges from 12 to 66 with higher scores indicating better social support (Wang et al., 2015).

#### Resilience

Resilience was assessed using the Connor-Davidson Resilience Scale (*CD-RISC*) (Connor and Davidson, 2003). The self-report questionnaire measures the degree of resilience in patients bipolar disorder, which the authors defined as “a positive individual characteristic that acquires the meaningful of life.” The Chinese version of the CD-RISC comprises 25 items. Each item uses a 5-level rating, ranging from 0 (*never*) to 4 (*always*). The total score ranges from 0 to 100, and a greater score indicates increased quality of resilience (Xian-Yun et al., 2010).

#### Suicidal Ideation

The Beck Scale for Suicide Ideation (*BSI*) (Beck et al., 1979) was used to measure the level of suicidal ideation. The BSI consisted of 19 items and was divided into two parts: suicidal ideation and suicidal behavior. Each item uses a 3-level rating from 0 to 2, and higher scores indicated stronger suicidal ideation. The first 5 items in the BSI were used to assess suicidal ideation among participants.

### Statistical Analysis

Analyses were conducted using Statistical Package for the Social Sciences (*SPSS*) version 25.0 and IBM AMOS version 23.0 (SPSS Inc., Chicago, IL, United States). Means and standard deviations (*SD*) were used to describe descriptive variables, and numbers (*n*) and percentages (*%*) were used for categorical variables. The normality of all data were test by the Shapiro-Wilk test. The mean differences in the demographic and clinical variables in the patient and HC groups were compared using the chi-square (*χ^2^*), one-way analysis of variance (*ANOVA*) followed by Bonferroni *post-hoc* tests or Mann-Whitney *U* tests. Partial correlation analyses were used to quantify potential bivariate associations among the FAD, SSRS, CD-RISC, BSI and FAST after controlling for HDRS-17 and YMRS scores, which are factors reported in our previous studies that potentially influence cognitive and psychosocial functioning (Lin et al., 2020). Several hierarchical regression analysis models were used to investigate those variables that could be predictors of psychosocial functioning. Total FAST scores and each dimension were introduced as the dependent variable in each model. The HDRS-17 and YMRS scores were entered in the first block as independent variables. The scores of the subscales of the FAD and SSRS and the BSI and CD-RISC scores were introduced in the second block as independent variables using a stepwise method. All the values of variance inflation factor (*VIF*) <10 and the values of tolerance >0.1 were considered adequate for the tests for multicollinearity. All statistics were tested using two-tailed comparisons and the 0.05 significance criterion.

**FIGURE 1:**
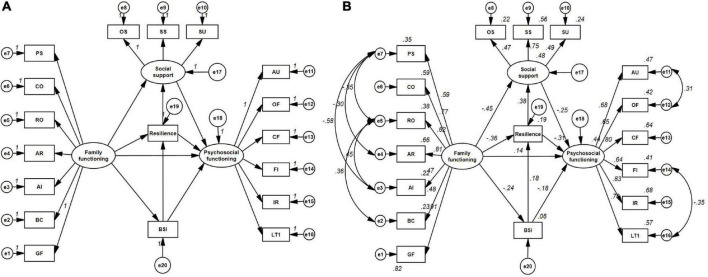
The original model (A) and final model (B). PS, Problem Solving; CO, Communication; RO, Roles; AR, Affective Responsiveness; AI, Affective Involvement; BC, Behavior Control; GF, General Functioning; OS, Objective Support; SS, Subjective Support; SU, Support Utilization; AU, Autonomy; OF, Occupational Functioning; CF, Cognitive Functioning; FI, Financial Issue; IR, Interpersonal Relationships; LT, Leisure Time; BSI, Beck Scale for Suicide Ideation.

The mediating effects in the hypothesis were examined using a structural equation model (*SEM*) constructed by AMOS 23.0. According to our previous study, family functioning could predict psychosocial functioning in BD patients, and social support had predicting and moderating effects on family functioning and psychosocial functioning (Dou et al. under review). Resilience mediates family adaptability and well-being and predicts psychosocial functioning, and this effect is strengthened by the inclusion of social support (Kukihara et al., 2020; Wang et al., 2021). Furthermore, the impairment of family functioning may lead to suicidal ideation, thereby impairing the psychosocial functioning of BD patients (Goldstein et al., 2009; Berutti et al., 2016). The original model is shown in Figure 1A.

Maximum likelihood estimation, including Chi-square/degree of freedom (*χ^2^/df*), incremental fit index (*IFI*), comparative fix index (*CFI*) and root mean square error of approximation (*RMSEA*), was used to test the satisfaction of each model. The model was deemed to have an acceptable fit when every path coefficients was significant (*p*-values <0.05); Chi-square/degree of freedom (*χ^2^/df*) was less than 2; root mean square error of approximation (*RMSEA*) was less than 0.08; and goodness-of-fit index (*GFI*), comparative fit index (*CFI*), normed fit index (*NFI*) and the Tacker-Lewis Index (*TLI*) were greater than or equal to 0.90 (McDonald and Ho, 2002). The standard estimate for the effects (direct, indirect, and total) was used to determine the connection between the observed and latent variables. Materials and analysis code for this study are available by emailing the corresponding author.

## Results

### Descriptive Analyses

The BD and HC groups did not show significant differences in most of the demographic variables, except for occupational status, educational level and WHR (*p* < 0.05, see Table 1). As expected, significant differences (*p* < 0.001) in the HDRS-17 and YMRS scores were noted the BD and HC groups.

As shown in Table 2, patients with BD rated significantly worse in family functioning and all psychosocial functioning areas than healthy individuals (all *p-*values <0.05), except for behavior control of the FAD (*p* = 0.107). For social support, significant differences in the total score of the SSRS (*p* = 0.018) and the subscale of subjective support (*p* = 0.003) were noted between the BD and HC groups, whereas no differences were observed in the subscales of objective support and support utilization between these two groups (all *p*-values >0.05). Furthermore, the BSI and CD-RISC scores in BD patients were lower than those in healthy individuals (all *p*-values <0.001).

**TABLE 2:** Comparisons on the family functioning, psychosocial functioning, social support, resilience and suicidal ideation between patients with bipolar disorder and healthy controls.

Variables	Patients with bipolar disorder (*n* = 246)		Healthy controls (*n* = 69)		ANOVA	
			
	*Mean*	*SD*	*Mean*	*SD*	*F*	*p*
**The FAD^#^**						
Total score of the FAD	142.91	18.87	130.99	20.03	1.045	<0.001**
Problem solving	13.19	3.30	11.83	2.72	3.048	0.002**
Communication	21.83	4.02	19.62	4.16	0.780	<0.001**
Roles	25.90	4.19	24.39	4.50	0.462	0.010*
Affective responsiveness	14.96	2.98	13.88	3.07	0.497	0.007**
Affective involvement	17.57	3.29	16.46	2.44	2.447	0.005**
Behavior control	21.55	3.19	20.86	2.98	2.309	0.107
General functioning	27.92	5.42	24.12	5.40	0.003	<0.001**
**The FAST^#^**						
Total score of FAST	16.52	14.43	7.75	8.69	18.082	<0.001**
Autonomy	2.40	2.46	1.42	1.71	12.839	<0.001**
Occupational functioning	3.88	4.39	1.57	2.83	30.312	<0.001**
Cognitive functioning	3.35	3.63	1.64	2.28	17.186	<0.001**
Financial issue	1.45	1.78	0.59	1.18	28.464	<0.001**
Interpersonal relationships	4.15	4.21	1.84	2.70	14.065	<0.001**
Leisure time	1.33	1.63	0.72	1.10	19.595	<0.001**
**The SSRS^#^**						
Total score of SSRS	34.72	9.29	37.65	8.01	2.781	0.018*
Objective support	8.31	3.88	8.49	2.85	2.019	0.716
Subjective support	19.24	5.88	21.57	5.42	0.881	0.003**
Support utilization	7.18	2.36	7.59	1.96	2.079	0.179
**The CD-RISC^#^**	55.61	23.13	67.87	17.91	5.319	<0.001**
**The BSI^#^**	6.25	5.54	1.93	2.07	9.996	<0.001**

*ANOVA, Analysis of Variance; SD, Standard deviation; FAD, Family Assessment Device; FAST, Functioning Assessment Short Test; SSRS, Social Support Rating Scale; CD-RISC, Connor-Davidson Resilience Scale; BSI, Beck Scale for Suicide Ideation.*

*^#^Higher scores of the FAD, FAST, and BSI scales indicate worse family and psychosocial functioning, and stronger suicidal ideation, respectively.*

*^#^Higher scores of the SSRS and CD-RISC indicate better social support and resilience, respectively.*

*p < 0.05. **p < 0.01.

### Correlational Analyses

As shown in Table 3, the FAD total score was positively associated with the total and subscale scores of the FAST (*r* range from 0.16 to 0.35, all *p*-values <0.05), whereas the FAST total score was positively correlated with the total and subscale scores of the FAD (*r* range from 0.13 to 0.30, all *p*-values <0.05) with the exception of affective involvement (*r* = 0.09, *p* = 0.16). In addition, significantly negative correlations were noted between the FAD and the SSRS, CD-RISC and BSI scores (*r* range from −0.15 to −0.42, all *p*-values <0.05) in patients with bipolar disorder. In addition, the FAST scores were negatively related to the SSRS, CD-RISC and BSI scores (*r* range from −0.15 to −0.46, all *p*-values <0.05) in the bipolar group. Furthermore, the SSRS was positively correlated with the CD-RISC and BSI scores (*r* = 0.39, *p* < 0.001; *r* = 0.15, *p* = 0.02; respectively), and the CD-RISC was also significantly associated with the BSI (*r* = 0.21, *p* = 0.001) in patients with bipolar disorder.

**TABLE 3:** The partial correlations^#^ among family functioning, psychosocial functioning, social support, resilience and suicidal ideation in patients with bipolar disorder.

Variables	FAD								FAST							SSRS				CD-RISC	BSI
	(1)	(2)	(3)	(4)	(5)	(6)	(7)	(8)	(9)	(10)	(11)	(12)	(13)	(14)	(15)	(16)	(17)	(18)	(19)	(20)	(21)
**(1) FAD**	1																				
(2) PS ^1^	0.45**	1																			
(3) CO ^1^	0.76**	0.50**	1																		
(4) RO ^1^	0.76**	0.02	0.38**	1																	
(5) AR ^1^	0.81**	0.27**	0.60**	0.54**	1																
(6) AI ^1^	0.58**	−0.20**	0.20**	0.65**	0.45**	1															
(7) BC ^1^	0.65**	0.02	0.28**	0.62**	0.46**	0.46**	1														
(8) GF ^1^	0.89**	0.53**	0.69**	0.52**	0.71**	0.39**	0.42**	1													
**(9) FAST**	0.30**	0.29**	0.29**	0.15*	0.22**	0.09	0.13*	0.30**	1												
(10) AU ^2^	0.20**	0.20**	0.18*	0.13*	0.08	0.06	0.09	0.22**	0.77**	1											
(11) OF ^2^	0.16*	0.19**	0.11	0.13*	0.04	0.04	0.13*	0.21	0.82**	0.61**	1										
(12) CF ^2^	0.24**	0.24**	0.25**	0.06	0.20**	0.11	0.12	0.24**	0.81**	0.53**	0.52**	1									
(13) FI ^2^	0.24**	0.18**	0.26**	0.14*	0.21**	0.09	0.03	0.24**	0.61**	0.43**	0.39**	0.43**	1								
(14) IR ^2^	0.35**	0.29**	0.34**	0.16*	0.31**	0.11	0.09	0.37**	0.82**	0.53**	0.52**	0.56**	0.48**	1							
(15) LT ^2^	0.21**	0.28**	0.23**	0.04	0.18**	−0.03	0.11	0.20**	0.69**	0.45**	0.45**	0.61**	0.25**	0.57**	1						
**(16) SSRS**	−0.42**	−0.18**	−0.34**	−0.30**	−0.40**	−0.24**	−0.25**	−0.36**	−0.30**	−0.21**	−0.15*	−0.27**	−0.13*	−0.35**	−0.29**	1					
(17) OS ^3^	−0.21**	−0.11	−0.12	−0.17**	−0.22**	−0.10	−0.18**	−0.15*	−0.15*	−0.08	−0.08	−0.15	0.02	−0.16*	−0.20**	0.69**	1				
(18) SS ^3^	−0.38**	−0.16*	−0.33**	−0.27**	−0.37**	−0.20**	−0.20**	−0.34**	−0.28**	−0.22**	−015*	−0.21**	−0.17**	−0.32**	−0.23**	0.88**	0.36**	1			
(19) SU ^3^	−0.32**	−0.10	−0.29**	−0.23**	−0.26**	−0.25**	−0.16*	−0.27**	−0.23**	−0.12	−0.10	−0.24**	−0.14*	−0.26**	−0.22**	0.53**	0.12	0.33**	1		
**(20) CD-RISC**	−0.33**	−0.23**	−0.33**	−0.17*	−0.27**	−0.12	−0.21**	−0.28**	−0.46**	−0.31**	−0.33**	−0.36**	−0.22**	−0.43**	−0.41**	0.38**	0.16*	0.35**	0.33**	1	
**(21) BSI**	−0.15*	−0.15*	−0.16*	0.01	−0.13*	−0.04	−0.10	−0.17**	−0.27**	−0.18**	−0.18**	−0.29**	−0.15*	−0.20**	−0.23**	0.15*	0.03	0.17**	0.10	0.21**	1

*FAD, Family Assessment Device; FAST, Functioning Assessment Short Test; SSRS, Social Support Rating Scale; CD-RISC, Connor-Davidson Resilience Scale; BSI, Beck Scale for Suicide Ideation; PS, Problem Solving; CO, Communication; RO, Roles; AR, Affective Responsiveness; AI, Affective Involvement; BC, Behavior Control; GF, General Functioning; AU, Autonomy; OF, Occupational Functioning; CF, Cognitive Functioning; FI, Financial Issue; IR, Interpersonal Relationships; LT, Leisure Time; OS, Objective Support; SS, Subjective Support; SU, Support Utilization.*

*^1^ family functioning; ^2^ psychosocial functioning; ^3^ social support.*

*^#^The 17-item Hamilton Depression Rating Scale and Young Mania Rating Scale scores were controlled as covariant variables.*

*p < 0.05, **p < 0.01.

### Hierarchical Regression Analyses

Table 4 demonstrates that resilience had a predictive effect on the total and subscale scores of the FAST (β ranging from −0.171 to −0.393, all *p*-values <0.05). Hierarchical regression analysis revealed that the significant predictors of the total FAST score included the HDRS-17 score (β = 0.216, *p* = 0.001), the FAD score (β = 0.139, *p* = 0.021), the CD-RISC score (β = −0.393, *p* < 0.001) and the BSI score (β = −0.131, *p* = 0.034), which explained 36.7% of the variance (*R*^2^ = 0.367, *F* = 20.701, *p* = 0.034). The HDRS-17 also predicted cognitive functioning (β = 0.198, *p* = 0.005), financial issues (β = 0.201, *p* = 0.009) and interpersonal relationships (β = 0.268, *p* < 0.001).

**TABLE 4:** Results of predicting effects of family functioning, social support, suicidal ideation, and resilience on psychosocial functioning.

Variables	Total score of FAST		Autonomy		Occupational functioning		Cognitive functioning		Financial issue		Interpersonal relationships		Leisure time	
	β	*p*	β	*p*	β	*p*	β	*p*	β	*p*	β	*p*	β	*p*
HDRS-17	0.216	0.001**	0.132	0.085	0.068	0.370	0.198	0.005**	0.201	0.009**	0.268	<0.001**	0.050	0.498
YMRS	0.025	0.701	0.044	0.559	0.024	0.750	0.067	0.338	0.023	0.759	0.018	0.777	−0.034	0.638
Age	–	–	–	–	−0.199	0.003**	–	–	–	–	–	–	–	–
Gender	–	–	–	–	–	–	–	–	−0.163	0.012*	–	–	–	–
The FAD	0.139	0.021*	–	–	–	–	–	–	0.158	0.022*	0.142	0.023*	–	–
The SSRS	–	–	–	–	–	–	–	–	–	–	−0.197	0.003**	−0.154	0.029*
The CD-RISC	−0.393	<0.001**	−0.343	<0.001**	−0.297	<0.001**	−0.318	<0.001**	−0.171	0.014*	−0.301	<0.001**	−0.350	<0.001**
The BSI	−0.131	0.034*	–	–	−0.174	0.013*	−0.220	0.001**	–	–	–	–	−0139	0.030*
**Goodness of fit of the model**														
R^2^	0.367		0.154		0.171		0.277		0.156		0.401		0.232	
F	0.034*		<0.001**		0.013*		<0.001**		0.022*		0.023*		0.030*	
p	20.701		13.353		9.394		20.526		8.569		23.793		13.335	
VIF ^8^(highest)	1.398		1.048		1.180		1.394		1.393		1.476		1.294	
Tolerance (lowest)	0.716		0.954		0.848		0.717		0.718		0.677		0.773	

*FAST, Functioning Assessment Short Test; HDRS-17, the 17-item Hamilton Depression Rating Scale; YMRS, Young Mania Rating Scale; FAD, Family Assessment Device; SSRS, Social Support Rating Scale; CD-RISC, Connor-Davidson Resilience Scale; BSI, Beck Scale for Suicide Ideation; VIF, variance inflation factor.*

*p < 0.05, **p < 0.01.

The FAD predicted the dimensions of financial issues (β = 0.158, *p* = 0.022) and interpersonal relationships (β = 0.142, *p* = 0.023), and interpersonal relationships (β = −0.197, *p* = 0.003) and leisure time (β = −0.154, *p* = 0.029) were predicted by the SSRS. The significant predictor of the total FAST score, occupational functioning, cognitive functioning and leisure time was BSI (β ranging from −0.131 to −0.220, all *p-*values <0.05). Interestingly, age (β = −0.199, *p* = 0.003) was predictive of occupational functioning, and financial issues were predicted by gender (β = −0.163, *p* = 0.012).

### Mediation Model

In the original mediation model (see Figure 1A), the SSRS, CD-RISC and BSI scores were included as mediators of the relationship between the FAD and FAST scores. The final model demonstrated acceptable fit: GFI = 0.916, NFI = 0.901, TLI = 0.944, CFI = 0.956, RMSEA = 0.054. In addition, the Chi-square test (*χ*^2^ = 207.259, *p* < 0.001) and Chi-square/degree of freedom (*χ^2^/df* = 1.713) were statistically significant after residuals correction. All beta values are standardized (see Figure 1B).

As presented in the final models (Figure 1B and Table 5), the direct effect between family functioning and psychosocial functioning was 0.137 after correction. The indirect effect through social support, resilience and suicidal ideation was calculated as follows:

Path 1. Family functioning → social support → psychosocial functioning

β=-0.454×-0.250=0.114

Path 2. Family functioning → resilience → psychosocial functioning

β=-0.359×-0.314=0.113

Path 3. Family functioning → suicidal ideation → psychosocial functioning

β=-0.236×-0.185=0.044

Path 4. Family functioning → resilience → social support →psychosocial functioning
Combined β = −0.359 × 0.375 × −0. 250 = 0.034
Path 5. Family functioning → suicidal ideation → resilience → psychosocial functioning
Combined β = −0.236 × 0.177 × −0. 314 = 0.013
Path 6. Family functioning → suicidal ideation →resilience → social support → psychosocial functioning
Combined β = −0.236 × 0.177 × 0.375 × −0. 314 = 0.005

**TABLE 5:** Standardized direct, indirect and total effects of family functioning on psychosocial functioning.

Exogenous variables	Endogenous variables	Standardized path coefficient		*C.R.*	*SE*	*SDE*	*SIE*	*STE*	*SMC*
		β	95% CI						
Family functioning	Social support	−0.454	[−0.607, −0.305]	−4.602**	0.037	−0.454**	−0.150**	−0.604**	0.483
Resilience		0.375	[0.209, 0.523]	4.214**	0.007	0.375**		0.375**	
Family functioning	Resilience	−0.359	[−0.461, −0.246]	−5.710**	0.296	−0.359**	−0.042**	−0.400**	0.190
Suicidal ideation		0.177	[0.054, 0.290]	2.961**	0.249	0.177**		0.177**	
Family functioning	Suicidal ideation	−0.236	[−0.360, −0.111]	−3.641**	0.073	−0.236**		−0.236**	0.056
Family functioning	Psychosocial functioning	0.137	[−0.059, 0.316]	1.653	0.028	0.137	0.320**	0.457**	0.438
Social support		−0.250	[−0.566, −0.036]	−2.136*	0.106	−0.250*		−0.250*	
Resilience		−0.314	[−0.463, −0.119]	−4.094**	0.006	−0.314**	−0.094*	−0.408**	
Suicidal ideation		−0.185	[−0.283, −0.081]	−3.206**	0.017	−0.185**	−0.072**	−0.257**	

*C. R., critical ratio; SE, standardized error; SDE, standardized direct effect; SIE, standardized indirect effect; STE, standardized total effect; SMC, squared multiple correlation.*

*p < 0.05, **p < 0.01.

The final model demonstrated that psychosocial functioning was predicted indirectly (β = 0.323) by family functioning through social support (β = 0.114; combined β = 0.034), resilience (β = 0.113; combined β = 0.013) and suicidal ideation (β = 0.044; combined β = 0.005), explaining a total of 43.8% of the variance in psychosocial functioning (*R*^2^ = 0.438).

## Discussion

To the best of our knowledge, this is the first study to investigate the mediating role of social support, resilience and suicidal ideation on family and psychosocial functioning in BD patients using a structural equation model. The results demonstrated that bipolar patients rated worse family and psychosocial functioning than healthy population. Furthermore, family functioning, social support, resilience and suicidal ideation could significantly predict psychosocial functioning in bipolar patients. Notably, social support, resilience and suicidal ideation could indirectly mediate the effect of family functioning on psychosocial functioning in bipolar patients.

Patients with BD had significant family and psychosocial dysfunction compared to healthy controls, which was in line with previous studies (MacPherson et al., 2018; Shalev et al., 2019). Findings add to the research on family factors and worse courses in patients with BD and indicate that family and psychosocial functioning are obviously dysfunctional, which demands consideration in implementing interventions (MacPherson et al., 2018; Luo et al., 2020). Interestingly, no difference in behavior control was noted between BD patients and healthy controls. This finding is inconsistent with earlier research (Keenan-Miller et al., 2012; MacPherson et al., 2018), which may be related to the different participants and measurements. Keenan-Miller et al. (2012) assessed family functioning and social impairment based on The Family Adaptability and Cohesion Evaluation Scale II (*FACES-II*) and The Conflict Behavior Questionnaire (*CBQ*). Behavior control refers to the way family members maintain expectations for each other (MacPherson et al., 2018). It is not surprising that children and adolescents were given more care and expectations than adults in families and that worse behavior control may increase the likelihood of family aggression by children with affective mental disorders (Keenan-Miller et al., 2012; Shalev et al., 2019). Existing family therapies treatments, such as Family-focused treatment (FFT), Child- and family-focused cognitive behavior therapy (RAINBOW) and Multi-family psychoeducational groups (MFPG), combined with pharmacotherapy in patients with BD, are demonstrated to make significant effects on improving clinical symptoms, reducing family aggression and gaining in social support (Pavuluri et al., 2004; Carr, 2009; Fristad et al., 2015; Miklowitz and Chung, 2016). Furthermore, the systemic family therapy also could make significant improvements in psychosocial functioning through the potential effectiveness of family functioning (Carr, 2009). In future studies, comprehensive assessment (e.g., Behavioral control, affective responsiveness, affective involvement, and roles) and competency training should be provided to their families. Psychosocial functioning (all dimensions) in bipolar patients was worse than that in healthy, which is in line with other and our previous studies (Sanchez-Moreno et al., 2017; Lin et al., 2020). Clinical factors, including past psychotic history, the number of hospitalizations, comorbidities and episodes of depression, have been demonstrated to be related to psychosocial functioning in previous studies (Sanchez-Moreno et al., 2017). Therefore, it is essential to implement psychosocial interventions for patients with BD.

Family functioning could significantly predict global and six domain-specific psychosocial functioning in patients with BD, suggesting that family has an influence on deficient psychosocial functioning. As suggested in Miklowitz and Chung (2016) and Sullivan et al. (2012), mood symptoms might affect the family environment and psychosocial functioning. There was no significant change in the results after controlling for the HDRS-17 and YMRS scores. This finding was enriched in the study by MacPherson et al. (2018), which documented that family dysfunction exists even when patients with BD are in remission. A prior study explored the significant association between family burden and psychosocial functioning in first-episode and chronic psychosis, again supporting the idea that family functioning is related to psychosocial functioning in general rather than in BD specifically (Koutra et al., 2016). Thus, evidence-based psychosocial treatments (*EBTs*) and psychoeducational interventions for patients with BD combined with family strategies improve functioning and better promote the recovery of patients (Fristad and MacPherson, 2014).

Patients with BD reported less social support than the general population, especially subjective support, which is consistent with the article by Lei and Kantor (2021). A previous study illustrated that subjective support was correlated with the behavior and development of patients as a psychological perception of reality (Lu et al., 2021). The assessment of the patient’s satisfaction with being respected, understood, and supported in society is necessary when intervening in the social support of patients with bipolar disorder (Lei and Kantor, 2021). Although the measurements are inconsistent, the findings are consistent previous studies (Guerrero-Jimenez et al., 2021; Lei and Kantor, 2021) suggesting that social support is related to family functioning and psychosocial functioning. The absence of social support, especially through their families of origin (parents and/or primary caregiver), can trigger clinical symptoms (Owen et al., 2017; MacPherson et al., 2018). Hypomania results in the establishment of new social relationships, whereas exacerbated affective symptoms can break intimate connections (Owen et al., 2017), creating a vicious cycle. In the present research, social support mediates the relationship between family and psychosocial functioning, and it is further well documented that social support of patients can enhance the impact of family functioning in promoting patient recovery. Therefore, it is important to develop a targeted support service in objective and subjective social support to strengthen family functioning and facilitate psychosocial functioning recovery in patients with BD.

As expected, the association between family and psychosocial functioning could be predicted and mediated by resilience in BD patients. Consistent with previous studies, resilience is an essential factor affecting family functioning and psychosocial functioning (Walsh, 2003; Kim et al., 2013). The relationship between resilience and family resilience is bidirectional. Family resilience, which is a component of the developmental perspective of family functioning, extends the understanding of family functioning in adversity (Walsh, 2015). Resilience can improve the quality of life and well-being of patients and their family members by actively contributing to the construction of well-functioning families (Kukihara et al., 2020; Wang et al., 2021). The explanation of mediatory role resilience is well family adaptability, and effective communication results in greater resilience, which is associated with better mental well-being (Kukihara et al., 2020). Notably, the assessment and intervention of family functioning of patients should emphasize the reconstruction of their resilience in coping with the diversity and complexity of family processes (Walsh, 2003, 2015). Another finding that cannot be ignored is that resilience negatively predicts psychosocial functioning directly and indirectly through the positive effect of social support. Resilience and social support as protective factors have been found to reduce the occurrence of abuse-related behaviors in patients with dementia (Lídia et al., 2018). The mediating role of social support between resilience and quality of life was explored in breast cancer patients (Zhang et al., 2017). Our findings contribute to the mediating roles of resilience, social support and psychosocial functioning on family functioning in patients with bipolar disorder. Therefore, family strategies should be formulated to improve psychosocial functioning of patients, and emphasis should be placed on enhancing resilience while strengthening social support.

Interestingly, suicidal ideation was negatively associated with family functioning, and this relationship was also found in suicidal ideation and psychosocial functioning. Nevertheless, in contrast to our present study, previous studies indicated that a worse family environment predicted higher suicidal ideation or suicide attempts (Goldstein et al., 2009; Berutti et al., 2016). Goldstein et al. (2009) combined the Conflict Behavior Questionnaire (*CBQ*), *FACES-II*, and the Life Events Checklist (*LEC*) to measure the family environment in 446 bipolar youth patients, and the total sample in the study of Berutti et al. (2016) was relatively small (62 participants). Another explanation for this distinction is that the best family relationship is formed by taking the best possible care of families in Chinese households. This may cause an increase in the sense of hopelessness, which consequently enhances suicidal ideation (Kwok and Shek, 2010). Notably, suicidal ideation plays a mediatory role between family and psychosocial functioning in BD patients. The families of suicidal patients were characterized by higher divorce and separation and lower cohesion and adaptability, which led to psychosocial dysfunction (Goldstein et al., 2009). Suicidal ideation as a predictor of compromised functioning has long been established, and early identification and continuous intervention are required, especially in family and psychosocial functioning (Reinherz et al., 2006; Berutti et al., 2016).

## Limitation and Strengths

Several limitations should be acknowledged in the study. First, the assessment of family functioning was only determined from data collected *via* patients’ self-reports and did not include reports from intimate relatives. Parents, offspring and spouses in BD families, which play indivisible roles in the daily life of patients with BD, often demonstrate a lack of consistent reporting in family functioning (Shalev et al., 2019). Thus, further studies of families of patients with BD should be rated to provide a comprehensive assessment of their living status and family members. Second, although medication was described at baseline, we cannot exclude the effects of pharmacotherapy on changes in these variables. For instance, benzodiazepine could cause sedation and affect the assessment of neurocognitive performance and psychosocial functioning (Baandrup et al., 2017). Third, the research design is cross-sectional, and causal inferences between family functioning and psychosocial functioning cannot be made. Additionally, suicidal ideation is the long-term outcome of multiple factors, and recent suicidal ideation or the number of attempted suicides does not correlate with worse family functioning (Berutti et al., 2016). Therefore, longitudinal assessments should ideally be conducted, which could explore variations in the relationship among family functioning, suicidal ideation and psychosocial functioning with the timeline. To the best of our knowledge, this is the first study to investigate the mediating role of social support, resilience and suicidal ideation in family functioning and psychosocial functioning in BD patients. Furthermore, our sample is better characterized by related clinical features, and the sample size is relatively large, which has yielded better results in prior studies.

## Conclusion

The present study notes significant family and psychosocial dysfunction, reduced social support and resilience, and a higher level of suicidal ideation in bipolar patients compared with healthy individuals. Associations were found among family functioning, psychosocial functioning, social support, resilience and suicidal ideation in bipolar patients. Social support, resilience and suicidal ideation could indirectly mediate the effect of family functioning on psychosocial functioning in bipolar patients. These findings suggest that clinical or community interventions for bipolar patients should be combined with family strategies and emphasize enhancing social support and resilience while paying attention to patients’ suicidal ideation, which might improve psychosocial functioning.

## Data Availability Statement

The raw data supporting the conclusions of this article will be made available by the authors, without undue reservation.

## Ethics Statement

The studies involving human participants were reviewed and approved by L2019ZSLYEC-021, Sun Yat-sen University. The patients/participants provided their written informed consent to participate in this study.

## Author Contributions

XL designed the study and wrote the protocol. WD and XL undertook the statistical analysis and wrote the first draft of the manuscript. XL, DL, and YZ revised the manuscript. XY, HF, LC, YZ, KZ, and DL managed the data collection and clinical evaluations. All authors contributed to and have approved the final manuscript.

## Conflict of Interest

The authors declare that the research was conducted in the absence of any commercial or financial relationships that could be construed as a potential conflict of interest.

## Publisher’s Note

All claims expressed in this article are solely those of the authors and do not necessarily represent those of their affiliated organizations, or those of the publisher, the editors and the reviewers. Any product that may be evaluated in this article, or claim that may be made by its manufacturer, is not guaranteed or endorsed by the publisher.
